# Induction of Skin-Derived Precursor Cells from Human Induced Pluripotent Stem Cells

**DOI:** 10.1371/journal.pone.0168451

**Published:** 2016-12-19

**Authors:** Yoriko Sugiyama-Nakagiri, Tsutomu Fujimura, Shigeru Moriwaki

**Affiliations:** Kao Corporation, Akabane Ichikai-Machi, Haga-gun Tochigi, Japan; University of Texas at Austin Dell Medical School, UNITED STATES

## Abstract

The generation of full thickness human skin from dissociated cells is an attractive approach not only for treating skin diseases, but also for treating many systemic disorders. However, it is currently not possible to obtain an unlimited number of skin dermal cells. The goal of this study was to develop a procedure to produce skin dermal stem cells from induced pluripotent stem cells (iPSCs). Skin-derived precursor cells (SKPs) were isolated as adult dermal precursors that could differentiate into both neural and mesodermal progenies and could reconstitute the dermis. Thus, we attempted to generate SKPs from iPSCs that could reconstitute the skin dermis. Human iPSCs were initially cultured with recombinant noggin and SB431542, an inhibitor of activin/nodal and TGFβ signaling, to induce neural crest progenitor cells. Those cells were then treated with SKP medium that included CHIR99021, a WNT signal activator. The induction efficacy from neural crest progenitor cells to SKPs was more than 97%. No other modifiers tested were able to induce those cells. Those human iPSC-derived SKPs (hiPSC-SKPs) showed a similar gene expression signature to SKPs isolated from human skin dermis. Human iPSC-SKPs differentiated into neural and mesodermal progenies, including adipocytes, skeletogenic cell types and Schwann cells. Moreover, they could be induced to follicular type keratinization when co-cultured with human epidermal keratinocytes. We here provide a new efficient protocol to create human skin dermal stem cells from hiPSCs that could contribute to the treatment of various skin disorders.

## Introduction

An important goal of current bioengineering efforts is to generate or reconstitute fully organized and functional organ systems from dissociated cells that have been propagated under defined tissue culture conditions. Stem cells (SCs) have the unique capacity to self-renew and to differentiate into the cell lineages that constitute their tissue of origin. Within the tissue, SCs reside in a specialized environment (termed the niche) and regulate their proliferation and differentiation to maintain and regenerate tissue [1–3].

Several kinds of SCs reside in the skin and they will be useful in the treatment of diseases and other skin problems such as burn wounds, chronic wounds and ulcers. Epithelial SCs reside in the bulge region of the hair follicle (HF), a specialized portion of the outer root sheath epithelium defined as the insertion site of the arrector pili muscle [4–7]. Bulge cells possess the ability to differentiate into all types of cutaneous epithelial cells including sebaceous glands and interfollicular epidermal keratinocytes [7]. Bulge cells contribute not only to the generation of new HFs with each hair cycle but also to the repair of the epidermis during wound healing [8–9]. To obtain epithelial SCs, human epidermal keratinocytes and epidermal SCs have been developed from induced pluripotent stem cells (iPSCs) [10–12]. Additionally, iPSCs-derived epidermal cells have the ability to reconstitute HFs with mouse dermal cells [12, 13].

On the other hand, despite the remarkable regenerative capacity of the skin dermis, adult dermal SCs have not yet been fully defined. Skin-derived precursor cells (SKPs) have been isolated as a self-renewing, multipotent precursor population from the dermis of rodents and humans [14]. SKPs can differentiate into neural and mesodermal progenies, including adipocytes, skeletogenic cell types and Schwann cells [14, 15]. In addition, SKPs display all the predicted properties of multipotent dermal SCs including dermal papilla hair induction properties in animal models [16].

Thus, SKPs are attractive tools for regenerating the skin dermis, however, isolating SKPs from human skin requires invasive surgical procedures and the cells isolated may have limited or variable abilities to proliferate and/or differentiate depending on the age of the donor and the culture conditions. Mesenchymal stem cells (MSCs), defined as cells that self-renew and are able to give rise to multiple mesenchymal tissues, also have the same problems. Therefore, there have been many studies that generated MSCs from pluripotent stem cells [17]. Therefore, the ability to generate significant numbers of SKPs from pluripotent SCs would be a valuable source of dermal SCs to generate full thickness skin.

In this report, we provide an efficient induction protocol of SKPs from human iPSCs. The human iPSC-derived SKPs (hiPSC-SKPs) express several genes and proteins that have been previously reported to be expressed by human SKPs [14]. As for their differentiation potential, hiPSC-SKPs can successfully differentiate into adipocytes, osteocytes and Schwann cells. In addition, in preliminary observations, hiPSC-SKPs were able to induce hair follicular keratinization when they were co-cultured with epidermal keratinocytes. These observations suggest that hiPSC-SKPs may facilitate the regeneration of human full thickness skin, including skin appendages, and should provide useful tools for new drug discovery for diseases of the skin and appendages.

## Materials and Methods

### Human iPSC culture

A human iPS cell line (201B7) generated by introducing four transcription factors (Oct3/4, Sox2, Klf4 and c-Myc) into human skin fibroblasts [18] was used in this study. The hiPSCs were cultured on inactivated SNL feeder cells using hiPSC medium containing 80% DMEM/F12 (3:1) medium (Sigma Aldrich), 20% knockout serum replacement (Life Technologies), 100 μM non-essential amino acids (Sigma Aldrich), 2 mM L-glutamine (Life Technologies), 0.1 mM β-mercaptoethanol (Life Technologies), 50 units/ml penicillin and 50 μg/ml streptomycin (Life Technologies) and 10 ng/ml bFGF (Wako).

### Generation of hiPSC-SKPs

When hiPSC colonies reached 80–90% confluence, they were dissociated into small cell clumps by pipetting several times and were then plated on SNL feeder cells in hiPSC medium without bFGF, including 500 ng/ml noggin (R&D Systems) and 10 μM SB431542 (Tocris Bioscience). After 5 days of culture, the hiPSC medium was removed and the cells were washed with D-PBS, after which they were cultured for 3–5 days in SKPs medium containing DMEM/F12 (Life Technologies), 2% B27 supplement (Life Technologies), 100 units/ml penicillin and 100 μg/ml streptomycin (Life Technologies), 40 ng/ml bFGF (Wako), 20 ng/ml EGF (R&D Systems) and 3 μM CHIR99021 (Cayman Chemical). When cells reached 80% confluence, they were dissociated using Accutase cell detachment solution (BD Biosciences) and were subcultured in new dishes in SKPs medium without CHIR99021. Do as CHIR99021, 5 μM Wnt-C59 (Cellagen Technology), 5 μM DAPT (Cellagen Technology), 1 μM Hh-Ag1.5 (Cellagen Technology), 5 μM PD0325901 (Bio Vision) were treated for 3–5 days, respectively.

### Differentiation properties of hiPSC-SKPs

For adipogenic and osteogenic differentiation, cells were seeded at 3x10^4^ cells/cm^2^ and allowed to adhere overnight in SKPs medium. Adipogenic differentiation was induced for 2 weeks using medium consisting of MEM (Life Technologies), 15% rabbit serum (Sigma Aldrich), 0.45 nM IBMX (3-isobutyl-1-methylxanthine) (Sigma Aldrich), 2.07 μM insulin (Sigma Aldrich) and 100 nM dexamethasone (Sigma Aldrich); all media were changed every 3–4 days. The cells were stained with oil red-O solution (ScienCell Research Laboratories).

Osteogenic differentiation was induced for 2 weeks using medium consisting of MEM (Life Technologies), 10% fetal bovine serum (Hyclone Laboratories), 100 nM dexamethasone (Sigma Aldrich), 10 mM β-glucerophosphatase (Sigma Aldrich) and 50 μM L-ascorbic acid-2 phosphate (Sigma Aldrich); all media were changed every 3–4 days. Alkaline phosphatase (ALP) activity was determined using a Vector Blue Alkaline Phosphatase Substrate kit (Vector Laboratories).

For Schwann cell differentiation, cells were seeded at 0.5x10^4^ cells/cm^2^ on dishes coated with 0.02 mg/ml laminin (Sigma Aldrich) and 0.1 mg/ml poly-L-lysine (Sigma Aldrich) and were allowed to adhere overnight in SKPs medium. Cells were then cultured in Schwann cell differentiation medium consisting of DMEM/F12 (Life Technologies), N2 supplement (Life Technologies), 5 μM forskolin (Sigma Aldrich) and 50 ng/ml heregulin-1 β (Peprotech) for 3 weeks; all media were changed every 2–3 days. Immunofluorescent analysis was used to examine the expression of S100β (Sigma Aldrich).

#### Microarray analysis

RNAs were prepared from 3 sets each of 5 day (NC) and 9 day (iPS-SKPs) cells in culture using RNeasy (Qiagen). The RNA samples were hybridized to Affimetrix GeneChip Human Genome U133 plus 2.0 Arrays and were analyzed using an Affimetrix GeneChip system. Microarray data were analyzed using GeneSpring software (Agilent Technologies).

#### RNA extraction, RT-PCR and Real-Time Quantitative RT-PCR

Total RNAs were extracted using RNeasy (Qiagen). cDNAs were synthesized by reverse transcription of 1 μg total RNA using a high capacity RNA-to cDNA kit (Applied Biosystems). PCR analysis was performed with KOD DNA polymerase (Toyobo) using the primers listed in Table 1 and the conditions described below. PCR products were separated on 1.5% agarose gels containing SYBR Safe DNA Gel Stain (Life Technologies). PCR amplification was performed as follows: 94°C for 2 min, 25–35 cycles at 98°C for 10 sec, 60 or 63°C for 30 sec, and 68°C for 30 sec. Quantitative RT-PCR (qPCR) was performed using custom made TaqMan® probes (Applied Biosystems). Total RNAs were used for single-stranded cDNA synthesis using a High-Capacity cDNA Reverse Transcription Kit (Life Technologies). qPCR was performed using TaqMan Gene Expression Assays with a StepOnePlus™ Real-Time PCR System (Life Technologies). The specific probes and primers used for each target gene were: PPARG, Hs01115513_m1; RUNX2, Hs00231692_m1; COL1A1, Hs00164004_m1; ZIC1,Hs00602749_m1; PAX3, Hs00240950_m1; WLS, Hs01553062_m1;DKK1, Hs00931310_g1; AXIN2, Hs00383297_m1; LEF1, Hs01547250_m1; IGFBP-5, Hs00181213_m1 (Applied Biosystems). The expression of genes of interest was normalized with that of RPLP0 (ribosomal protein large P0, Hs99999902_m1) using a relative standard curve method for each target. An unpaired two-tailed t test was used to calculate whether differences between samples, across three technical replicates, were significant, or p<0.05.

**Table 1:** List of genes and primer sets used in the study.

Gene	Sense Primers (5'-3')	Anti-Sense Primers (5'-3')
GAPDH	cggagtcaacggatttggtcg	agccttctccatggtggtgaa
Oct-4	cgaaagagaaagcgaaccag	gtgaagtgagggctcccata
Nanog	cagaaggcctcagcacctac	gcctccaagtcactggcag
Snail	accgcctcgctgccaatgct	gtgcatcttgagggcaccca
Slug	catctttggggcgagtgagtcc	cccgtgtgagttctaatgtgtc
Twist	atgatgcaggacgtgtccag	tgccatcttggagtccagctc
Dermo-1	gcaagaagtcgagcgaagatg	ggcaatggcagcatcattcag
Nestin	cagcgttggaacagaggttg	gctggcacaggtgtctcaag
Sox9	gtcagccaggtgctcaaagg	acttgtaatccgggtggtcc
BMP-4	ttctgcagatgtttgggctgc	agagccgaagctctgcagag
Wnt-5a	ggatggctggaagtgcaatg	acacaaactggtccacgatc
Versican	acgatgcctactttgccacc	tagtgaaacacaaccccatcc
CD133	atggccctcgtactcggctc	cacgcggctgtaccacatag

### Immunofluorescence

Cells were fixed with 4% paraformaldehyde (Wako) for 15 min, permeabilized with 0.1% Triton X-100 for 5 min and blocked with 10% goat serum (Nichirei Bioscience) in PBS for 1 hr at room temperature (RT). Cells were incubated with primary antibodies for 2 hr at RT before incubation with appropriate secondary antibodies for 1 hr at RT, prior to counterstaining with 4’,6-diamidino-2-phenylindole (DAPI)(Dojindo Laboratories). Samples were observed using a Leica fluorescent microscope (Leica Microsystems). The primary antibodies used were: anti-nestin antibody (1:100; Millipore), anti-fibronectin antibody (1:100; Sigma-Aldrich), anti-αSMA antibody (1:100; Sigma-Aldrich), anti-S100β antibody (1:100; Sigma-Aldrich) and anti-trichohyalin antibody (1:5; Santa Cruz Biotechnology). Secondary antibodies used were: Alexa Fluor 488-conjugated goat anti-mouse (1:200) and Alexa Fluor 546-conjugated goat anti-rabbit (1:200, both from Life Technologies).

### Flow cytometric analysis

After cells were detached and collected, they were fixed and permeabilized with cytofix buffer for 20 min. After washing with Phosflow Perm/Wash buffer, cells were incubated for 30 min with the anti-nestin antibody conjugated with PerCP-CyTM5.5 and with the anti-fibronectin antibody conjugated with Alexa fluor 488 (both from BD Biosciences). Isotype-matched monoclonal antibodies (mAbs) were used as negative controls. In order to calculate the differentiation efficiency of iPSCs into SKPs, stained cells were analyzed using BD FACSVerse with FACSuite software (BD Biosciences)

### Organotypic assay imitating human HF-like epithelial- mesenchymal interactions

Human primary keratinocytes (J-TEC) were cultured in keratinocyte medium (J-TEC). Human primary fibroblasts (Kurabo) were cultured in DMEM (LifeTechnologies) including 5% fetal bovine serum (Hyclone Laboratories). Human dermal papilla cells (Cell Applications) were cultured in dermal papilla cell medium (Cell Applications). Four x 10^4^ cells of each cell type were mixed and cultured in 96-well-round-bottom assay plates (Cell Seed) containing 200 μl Amniomax C-100 medium (Life Technologies) with medium changes every 1–2 days. After 7 days in culture, cell aggregates were embedded in OCT compound (Tissue-Tek; Sakura Fine technical) and then frozen sections were stained.

## Results

### Generation of hiPSC-SKPs

**Fig 1:**
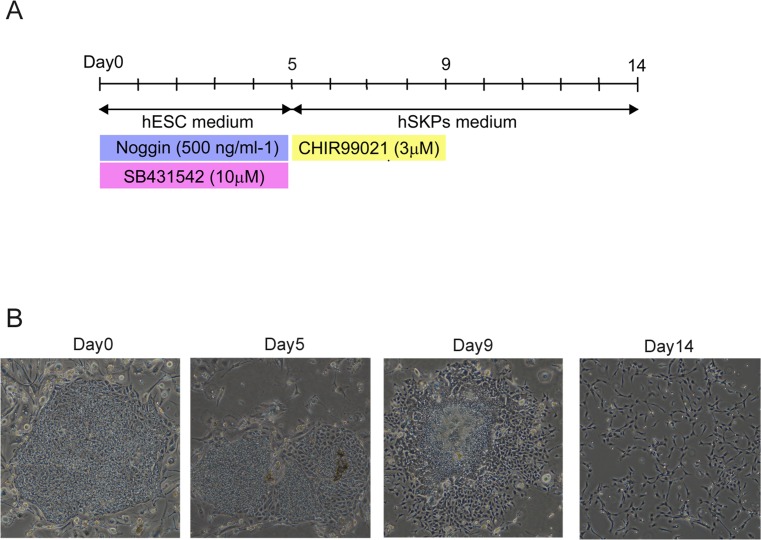
Generation of SKPs from hiPSCs. (A) Summary of the established protocol used to differentiate hiPSC-SKPs. Human iPSCs were cultured in initial differentiation medium with recombinant noggin and a TGF-β inhibitor (SB431542: SB). At day 5 of differentiation, the medium was changed to SKPs medium containing a WNT agonist (CHIR99021: CHIR) for 4 days. When the cells reached 80% confluence, they were dissociated and subcultured in new dishes in SKPs medium without CHIR. (B) Morphological characteristics of hiPSCs during differentiation.

**Fig 2:**
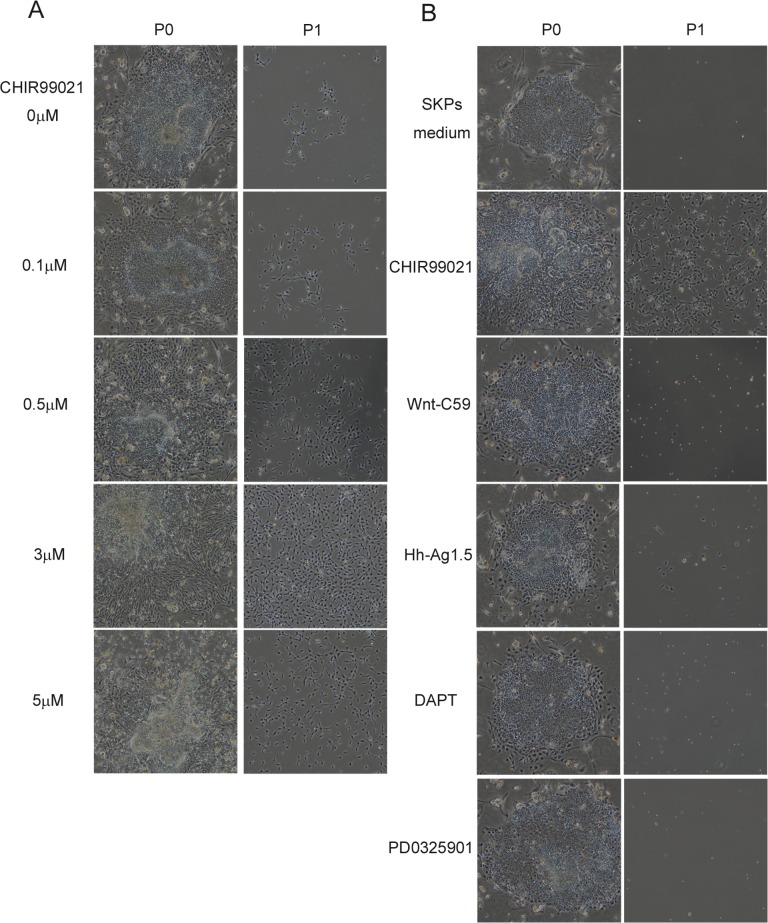
Human SKPs induction is driven by WNT activation. (A) Effective concentration of CHIR needed to induce hiPSC-SKPs. (B) Effects of modifiers used to induce hiPSC-SKPs. After noggin and SB treatment for 5 days, cells were cultured in SKPs medium with signal activators or inhibitors as noted for 4 days.

To generate SKPs from hiPSCs, we established a step-wise differentiation protocol in which hiPSCs were initially differentiated to the multipotent neural crest stage as precursor cells of SKPs (Fig 1A). Human iPSCs were treated for 5 days with human recombinant noggin (to inhibit BMP signaling) and SB431542 (SB) (to inhibit activin/nodal and TGFβ signaling) since inhibition of both signaling pathways promotes highly efficient neural induction from human PSCs [19–20]. The induction of SKPs was then initiated in SKPs medium including CHIR99021 (CHIR), which functions as an agonist of WNT signaling. Cells gradually migrated from the colonies and proliferated during induction. Cells were harvested after 4 days of induction, and were subsequently cultured in SKPs medium without CHIR. After 5 days, we obtained a sufficient number of cells, which we termed hiPSC-SKPs (Fig 1B). We then attempted to determine the most effective concentration of CHIR (with fixed conditions of noggin and SB) that could induce progenitor cells. The results revealed that CHIR successfully induced hiPSC-SKPs in a dose-dependent manner up to 3 μM, but higher concentrations of CHIR markedly inhibited the production of hiPSC-SKPs (Fig 2A). Moreover, none of the other modifiers tested was able to induce those cells (Fig 2B).

**Fig 3:**
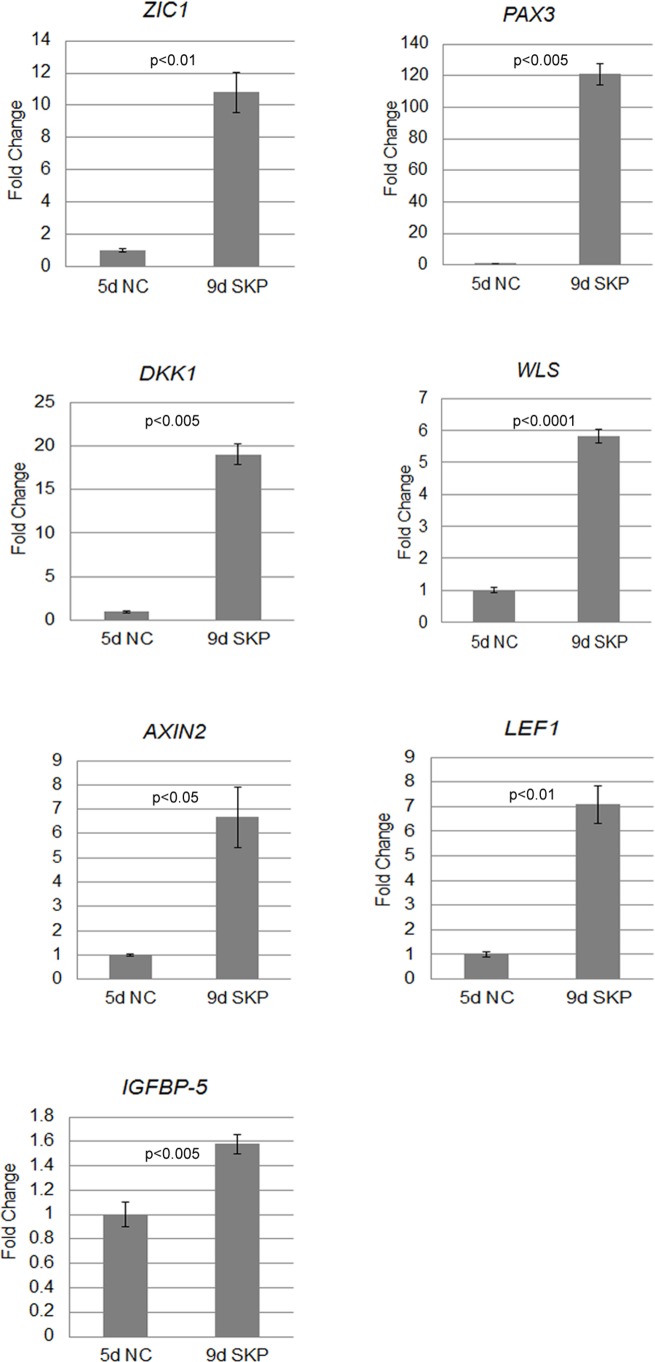
Gene expression analysis during hiPSC-SKPs differentiation. Quantitative real time PCR results showing changes between NC and iPSC-SKPs.

We next performed gene expression analysis to characterize which signals were regulated during the NC to iPSC-SKPs differentiation (Fig 3). The up-regulation of ZIC1 and PAX3, signals required for NC differentiation [21], confirmed that iPS cells were definitely induced to neural crest cell lineage during our induction method. Four days after CHIR exposure, AXIN2, DKK1, WLS and LEF1 were up-regulated. Thus, CHIR treatment induced endogenous WNT signaling during differentiation. In addition, IGFBP-5, a gene that contributes to bone formation and osteoblast differentiation, was up-regulated [22],

### Characterization of hiPSC-SKPs

**Fig 4:**
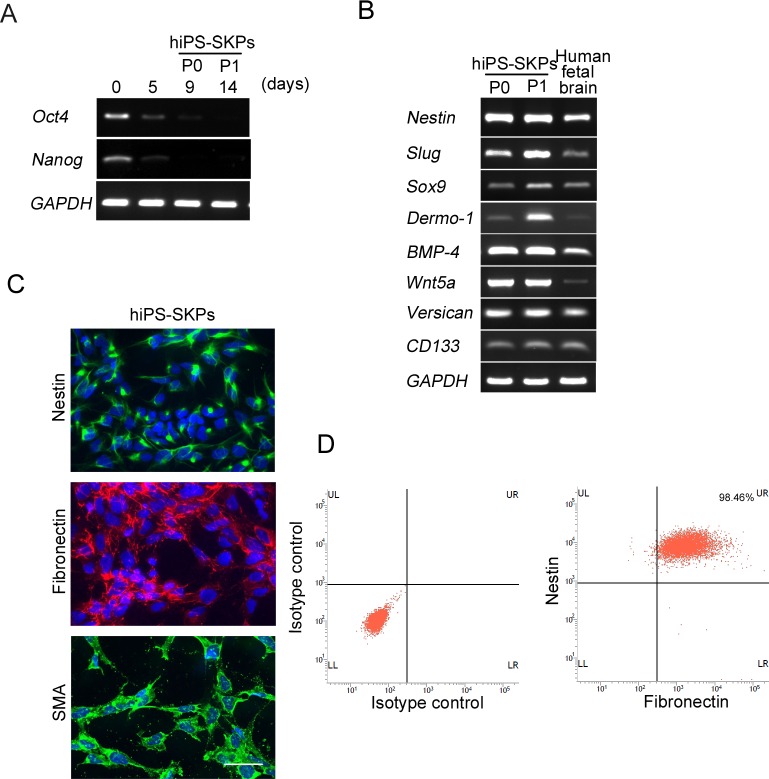
Molecular characterization of hiPSCs-derived SKPs. (A) RT-PCR analysis of genes expressed by hiPSCs, and (B) hSKPs in total RNAs from hiPSCs during differentiation. RNA from human fetal brain serves as a positive control, while GAPDH is used as a loading control. (C) Immunocytochemical analysis of nestin, fibronectin and α-SMA in hiPSCs-derived SKPs. Scale bar, 100 μm. (D) Flow cytometric analysis of hiPSCs-derived SKPs to detect the number of nestin and fibronectin positive hiPSC-SKPs.

In order to characterize hiPSC-SKPs, we examined their expression patterns of genes that have been well characterized for SKPs [14, 23]. RT-PCR analysis showed that hiPSCs markers, such as Oct4 and Nanog, were barely detected in differentiated hiPSC-SKPs (Fig 4A). hiPSC-SKPs expressed mRNA transcripts for Nestin, Slug, Sox9, Dermo-1, BMP4, Wnt5a and Versican (Fig 4B). Moreover, levels of Slug, Sox9 and Dermo-1 mRNA transcripts in hiPSC-SKPs increased after the cells were subcultured (Fig 4B). Immunocytochemistry showed that Nestin, Fibronectin and αSMA were also expressed in hiPSC-SKPs (Fig 4C). Furthermore, we monitored the expression of Nestin and Fibronectin in hiPSC-SKPs using flow cytometric analysis and found that 98.5% of those cells co-expressed Nestin and Fibronectin (Fig 4D).

### hiPSC-SKPs can differentiate into several cell types

**Fig 5:**
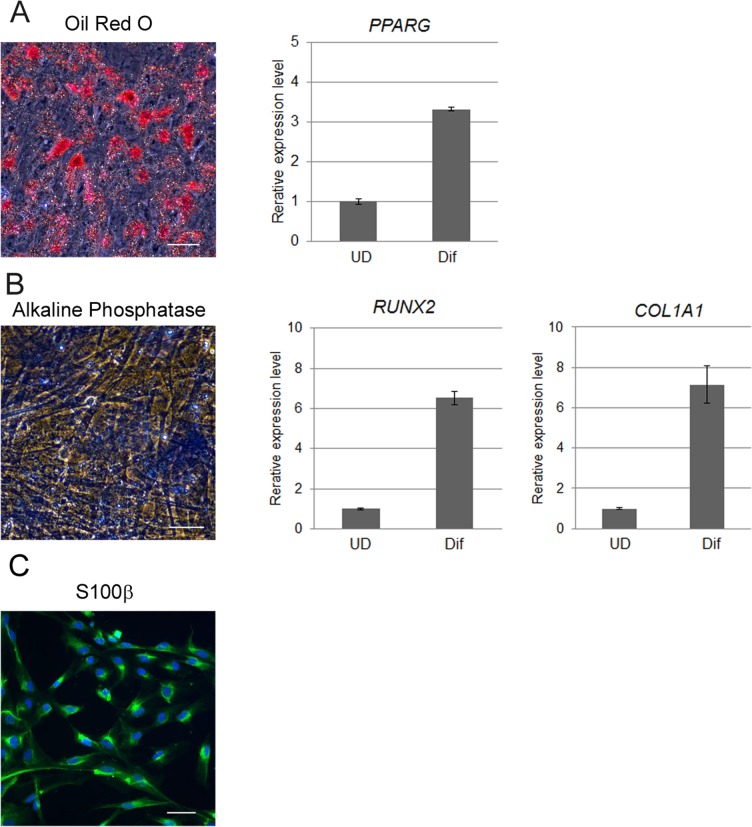
hiPSCs-derived SKPs display multipotency similar to traditionally isolated SKPs. (A) Adipogenic differentiation of hiPSC-SKPs was confirmed by Oil Red-O staining and the up-regulation of PPARγ gene expression by qPCR. (B) Osteogenic differentiation was confirmed by ALP staining and the up-regulation of RUNX2 and COL1A1 gene expression by qPCR. (C) Differentiated Schwann cell lineages were detected by S100β staining. Scale bars, 50 μm. UD: undifferentiated hiPSC-SKPs, Dif: differentiated hiPSC-SKPs.

To determine whether hiPSC-SKPs are also functionally similar to traditional SKPs, we examined the differentiation potentials of hiPSC-SKPs. hiPSC-SKPs were cultured in each differentiation medium for 2 or 3 weeks. Adipogenic differentiation was conducted in adipogenic differentiation medium. After 2 weeks of differentiation in that medium, the hiPSC-SKPs displayed significant lipid accumulation in the cytoplasm, which was positively stained with oil Red-O (Fig 5A). The mRNA expression level of an adipogenic gene, PPARγ, was up-regulated relative to undifferentiated hiPSC-SKPs. After 2 weeks of osteogenic differentiation, the expression of ALP, a popular marker of osteocytes, stained positive in hiPSC-SKPs (Fig 5B). Further, the mRNA expression levels of two osteogenic genes, RUNX2 and COL1A1, were up-regulated after differentiation. Schwann cell differentiation was promoted by the neural cell induction medium and cells expressed S100β after 3 weeks in culture (Fig 5C). These results show that hiPSC-SKPs are multipotent and are able to differentiate into adipogenic, osteogenic and neurogenic lineages the same as traditional SKPs.

### hiPSC-SKPs can induce follicular type keratinization

**Fig 6:**
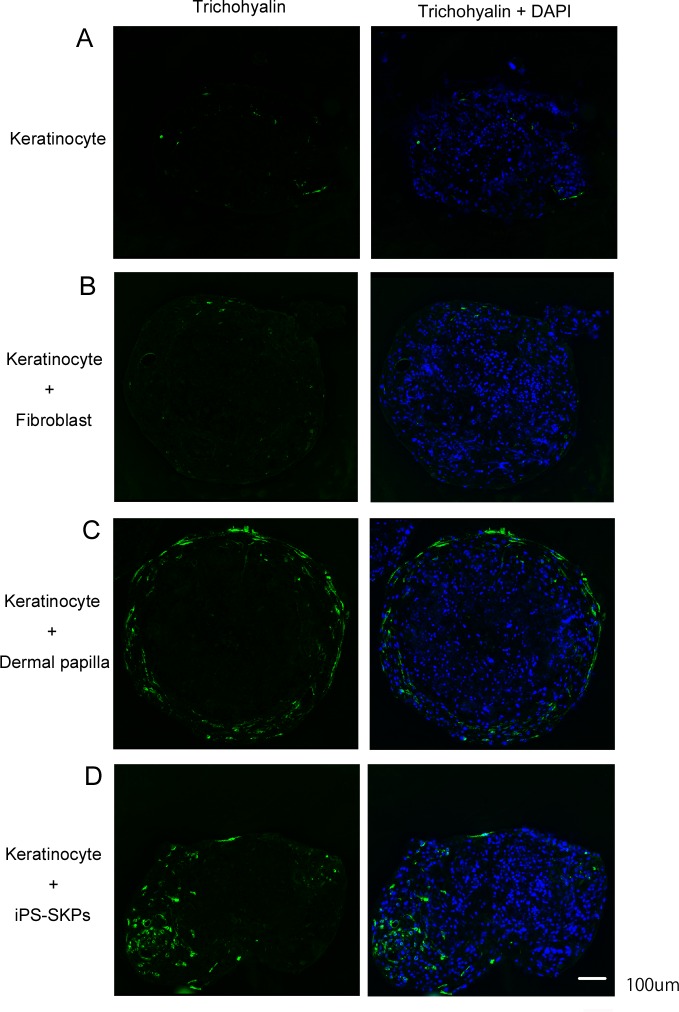
hiPSCs-derived SKPs display follicular mesenchymal -epithelial interactions in spheroid culture. Immunofluorescence staining of trichohyalin in human epidermal keratinocytes cultured in spheroid conditions (A), with human dermal fibroblasts (B), human dermal papilla cells (C) or hiPSC-SKPs (D) for 7 days. Scale bar, 100 μm.

Human epidermal keratinocytes and dermal fibroblasts, dermal papilla cells or hiPSC-SKPs were mixed and co-cultured in 96-well-round-bottom assay plates. After 7 days in culture, aggregates consisting of epidermal keratinocytes and hiPSC-SKPs expressed trichohyalin (Fig 6D), an HF specific protein, as did cell aggregates consisting of epidermal keratinocytes and dermal papilla cells (Fig 6C). In contrast, cell aggregates consisting of only epidermal keratinocytes (Fig 6A) or epidermal keratinocytes and dermal fibroblasts (Fig 6B) did not express or expressed only very low levels of trichohyalin.

## Discussion

In the present study, we developed a simple and efficient method to induce hSKPs from hiPSCs. The induction efficiency of this method is very high (over 95%) in a short period and the hiPSC-SKPs exhibit SKP characteristics under maintenance culture conditions.

SKPs were originally isolated as adult SCs capable of generating neural cell types from mammalian skin [14]. SKPs also display the functional properties predicted of dermal SCs, contributing to dermal maintenance, wound-healing and HF morphogenesis [23]. Thus, the ability to produce hiPSC-SKPs can provide an unlimited number of dermal SCs and should contribute to skin dermal regeneration that was lost due to injury or disease without surgical stress for any patients who need it including aged donors and those with various disorders.

In this study, hiPSC-SKPs exhibit many of the differentiation properties attributed to SKPs previously reported for multiple cell lineages including adipocytes, skeletogenic cell types and Schwann cells. Thus, our induction method can induce multipotent SCs similar to SKPs. However, hiPSC-SKPs also are able to differentiate into osteogenic cells unlike SKPs. An osteogenic differentiation property was reported in mSKPs that were formed from neonatal and adult fibroblasts in culture [24]. In addition, hiPSC-SKPs express IGFBP-5, a gene correlated with bone morphogenesis, unlike bona-fide SKPs (Fig 3). This characteristic is related to the osteogenic differentiation property. These facts suggest that hiPSC-SKPs have nearly equal quality, however, they differ somewhat from bona-fide SKPs. In future studies, we will define their differences more precisely to improve the safety of hiPSC-SKPs.

SKPs reside within the dermal papilla and dermal sheath of HFs. It has also been reported that SKPs can induce HF morphogenesis in rodents [16]. However, this HF generating property has not yet been demonstrated in human SKPs. In our present study, hiPSC-SKPs were able to induce trichohyalin expression when co-cultured with human keratinocytes. However, we could not detect HF morphogenesis in cell transplant experiments. It has been demonstrated that rodent dermal papilla cells can be removed from HFs and transplanted in their intact state into recipient skin, where they induce *de novo* HF development and hair growth [25–26]. However, isolated human dermal papilla cells have lost some intrinsic properties that are required for HF induction [27–28]. Thus, if human SKPs and/or iPS-SKPs have HF induction properties, it may be very difficult to demonstrate them in *in vivo* experiments. Our data show that hiPSC-SKPs express CD133, a dermal papilla marker, display follicular mesenchymal-epithelial interactions with human epidermal keratinocytes and induce trichohyalin expression in spheroid cultures *in vitro* (Fig 6). In another respect, cultured dermal cells, including dermal papilla and dermal sheath cells, are capable of being directed to adipogenic and osteogenic differentiation [29]. These findings suggest that hiPSC-SKPs might have similar properties to dermal papilla cells. Recent studies have demonstrated that some biological pathways are required for human HF induction properties and have succeeded in maintaining the intrinsic properties of HF regeneration [30–32]. These signal modifications to hSKPs or hiPSC-SKPs are probably also needed to acquire the HF induction abilities.

In conclusion, we have developed a simple protocol to generate hiPSC-SKPs. Our study provides a substantial contribution to the development of skin tissue regeneration, including the dermis and HFs, that are lost due to injury or diseases such as inherited disorders and androgenic alopecia.

## Abbreviations

iPSCs: induced pluripotent stem cells
SKPs: skin-derived precursor cells
hiPSC-SKPs: human iPSC-derived SKPs
SCs: stem cells
HF: hair follicle
CHIR: CHIR99021
MSCs: Mesenchymal Stem Cells
ALP: Alkaline Phosphatase
PBS: Phosphate Buffered Saline
RT: Room Temperature
IBMX: 3-isobutyl-1-methylxanthine
PCR: Polymerase Chain Reaction
qPCT: Quantitative Polymerase Chain Reaction
DAPI: 4',6-Diamidino-2-Phenylindole, Dihydrochloride
SB: SB431542
